# Supplementary material for: Transcriptomic Dynamics of Active and Inactive States of Rho GTPase MoRho3 in Magnaporthe oryzae
Source: J Fungi (Basel). 2022 Oct 11;8(10):1060. doi: 10.3390/jof8101060 (PMC9605073; doi:10.3390/jof8101060)
Supplement: Supplementary file 1 [file jof-08-01060-s001.zip › jof-1909100-supplementary.pdf]

Article

Transcriptomic Dynamics of Active and Inactive States of Rho GTPase MoRho3 in *Magnaporthe oryzae*

Qian Li [1,*,†], Xi Chen [2,†], Lianyu Lin [2,†], Lianhu Zhang [2,3], Li Wang [1], Jiandong Bao [2] and Dongmei Zhang [2,*]

1 Meishan Vocational Technical College, Ministerial and Provincial Joint Innovation Centre for Safety Production of Cross-Strait Crops, Fujian Agriculture and Forestry University, Fuzhou 350002, China
2 State Key Laboratory for Ecological Pest Control of Fujian and Taiwan Crops, College of Plant Protection, Fujian Agriculture and Forestry University, Fuzhou 350002, China
3 College of Agronomy, Jiangxi Agricultural University, Nanchang 330045, China
* Correspondence: lixianqian5220@163.com (Q.L.); zhangdongmei127@126.com (D.Z.)
† These authors contributed equally to this work.

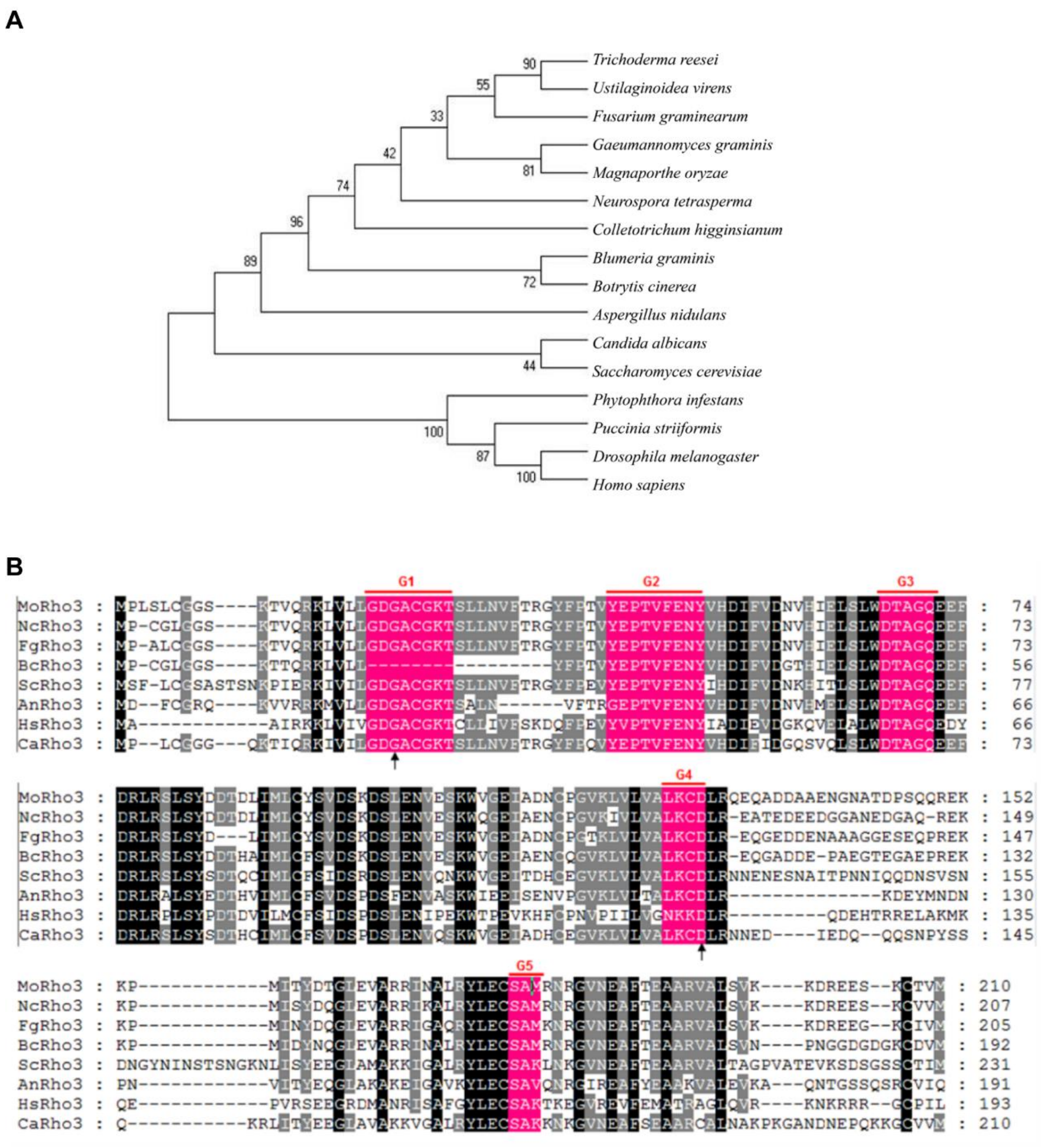

Figure S1 (A) Phylogenetic tree of Rho3 from fifteen species. **(B)** Amino acid sequence alignment of Rho3 from different species. Protein IDs: NcRho3 (XP_965740.1), FgRho3 (XP_011315782.1), BcRho3 (XP_001559261.1), ScRho3 (AJR37036.1), AnRho3 (XP_662386.1), HsRho3 (NP_786886.1), CaRho3 (XP_002418433.1).

A

Sample	Total reads	Aligned pairs	Aligned Left	Aligned Right	Concordant rate	Mapped Rate
**WT-1**	31643854	12070743	13583897	12648229	75.50%	82.90%
**WT-2**	27481018	10411795	11566748	10873734	75.00%	81.66%
**WT-3**	29008036	11071327	12318887	11589616	75.60%	82.42%
**CA_1**	25128312	9524108	10610783	9943999	74.70%	81.80%
**CA_2**	26300082	10235273	11369403	10710591	76.80%	83.95%
**CA_3**	21436846	8016529	9234958	8464881	73.80%	82.57%
**DN_1**	21526246	8317546	9380194	8749072	74.80%	84.22%
**DN_2**	28661096	11155136	12482728	11734388	75.50%	84.49%
**DN_3**	27440216	10458483	11683000	11015613	73.80%	82.72%

B

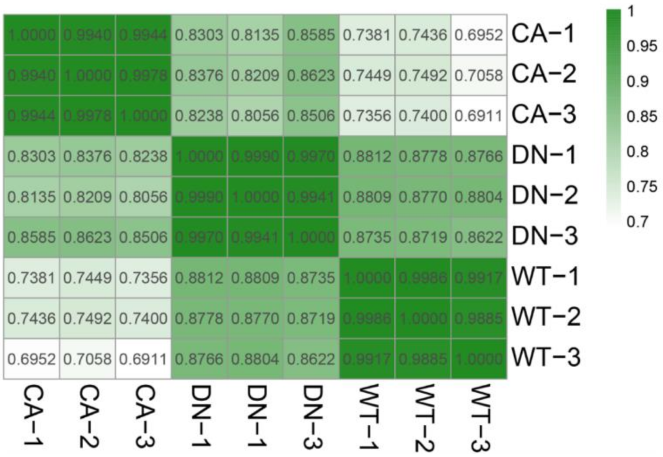

Figure S2 (A) Alignment result of Tophat. **(B)** Pairwise Pearson Correlation Coefficient of each two samples.

Table S1 qRT-PCR Primers used to validate RNA-seq reproducibility.

Name	Sequence	Description
Rho3qF	CTATGGGATACAGCAGGACAAG	Rho3
Rho3qR	GACCGAATAGCAGAGCATGA	
MGG_02378F	CCAGACTTGGAATGGGACTTT	Glutamate decarboxylase 1
MGG_02378R	GGATAGACAAGGCCGTACTTATG	
MGG_06888F	GCAAAGGGCTACGGTTACTT	Glutamine synthetase
MGG_06888R	CCAAAGCAGGTCTCCATGATAA	
MGG_15990F	CTCTTCAGCAGCTTCGTCATT	Hypothetical protein
MGG_15990R	CCGTTGACGACCACAAACA	
MGG_09261F	GAGCATGATGACCACCTTCA	Hypothetical protein
MGG_09261R	TTAGACACCACGGAAATCCTC	
MGG_04913F	GGACATCAGCTTCAACATGGA	CVNH domain-containing protein
MGG_04913R	GGTTGATATCGGCATCGTGAA	
MGG_16585F	CCCGTCCAGCCACTGTA	Hypothetical protein
MGG_16585R	ATCGCTGCAGACGCAGA	
MGG_10098F	GATGGTGGCGGATTGGAA	Kelch motif protein
MGG_10098R	TCGAGAACATCGGCAAAGAG	
MGG_00832F	GACGACGAGATGTTTGTGTTTG	Cytochrome P450
MGG_00832R	GGATGACGTGACTGTTGGT	
MGG_12763F	GACCAACACACCTGAGAACAC	Hypothetical protein
MGG_12763R	CGCATCATCGTCGTCATTGT	
MGG_07216F	GAGGGTCTGGCAAACATGAA	versicolorin reductase
MGG_07216R	CGTTGATCCATTCACTCTCCTC	
MGG_08535F	CTTTCACCGGCATCAACAAC	Hypothetical protein
MGG_08535R	GTGAAGAAGAAGGCCAGGAA	
MGG_12316F	CCACCTTTGAGGATGAGTACAA	GTP-binding protein EsdC
MGG_12316R	CGGTAGTACGAGGACAGGT	
MGG_07571F	TCACCATGACCCAGCTCTAT	LysM domain-containing protein
MGG_07571R	AACATGCACGCACATGTAGTA	
MGG_08989F	CCGTGGACACTCTTGACTTT	Adh short protein
MGG_08989R	CTCGGGCTGGAACTCTTTG	
MGG_14715F	GCAGGATAGCTGGGTTGTTA	Hypothetical protein
MGG_14715R	GCTCGTAGGTCACGTTGATAG	
MGG_08355F	CATCATCAACTACGCCTTCGAC	Hypothetical protein
MGG_08355R	GGCACTGCTTGCCGTAAA	
MGG_10275F	GCTCGACTACACCGTCTACTA	Sphingomyelin phosphodiesterase
MGG_10275R	CTTGACCGAGTACAGCTTCTG	
MGG_16714F	TCTCCTGCGCTGCACAA	Hypothetical protein
MGG_16714R	ATCCGTCAAGATCGCACTCC	
MoTubQRTF	TCCGTGGAAAGGTTTCCATG	beta-tubulin
MoTubQRTR	ATCCACTCGACGAAGTACGA	

Table S2 Quality and filter result of RNA-seq sequencing reads.

Sample	Raw Reads	Clean Reads	Raw Base(G)	Clean Base(G)	Effective Rate(%)	Error Rate(%)	Q20(%)	Q30(%)	GC Content(%)
WT-rep1	16,616,855	15,821,927	4.99	4.75	95.22	0.02	97.5	94.22	56.28
WT-rep2	14,433,110	13,740,509	4.33	4.12	95.2	0.02	97.69	94.6	55.89
WT-rep3	15,274,575	14,504,018	4.58	4.35	94.96	0.02	97.69	94.63	55.95
CA-rep1	12,840,099	12,564,156	3.85	3.77	97.85	0.02	97.22	93.8	54.86
CA-rep2	13,387,146	13,150,041	4.02	3.94	98.23	0.02	97.35	94.06	55.56
CA-rep3	10,905,512	10,718,423	3.27	3.22	98.28	0.03	96.38	92.06	55.78
DN-rep1	10,947,764	10,763,123	3.28	3.23	98.31	0.02	97.06	93.54	55.58
DN-rep2	14,598,478	14,330,548	4.38	4.3	98.16	0.02	97.16	93.75	55.45
DN-rep3	13,994,549	13,720,108	4.2	4.12	98.04	0.02	97.24	93.86	55.3
